# α-Synuclein: The Long Distance Runner

**DOI:** 10.1111/bpa.12046

**Published:** 2013-04-16

**Authors:** Sonia George, Nolwen L Rey, Nicole Reichenbach, Jennifer A Steiner, Patrik Brundin

**Affiliations:** 1 Neuronal Survival Unit, Wallenberg Neuroscience Center, Lund UniversityLund, Sweden; 2 Van Andel Research Institute, Center for Neurodegenerative ScienceGrand Rapids, MI

**Keywords:** α-Synuclein, Lewy pathology, Parkinson's disease, prion disease, prion-like aggregation, templated misfolding

## Abstract

Parkinson's disease is characterized by α-synuclein pathology in the form of Lewy bodies and Lewy neurites. Braak *et al* described the spatial and temporal spread of α-synuclein pathology in Parkinson's disease. Recent experimental studies have demonstrated that α-synuclein can transfer from cell to cell. In this review, we highlight the involvement of α-synuclein in Parkinson's disease and in Braak's staging of Parkinson's disease pathology. We discuss whether a prion-like mechanism of α-synuclein spread might contribute to Parkinson's disease pathology. We describe recent studies investigating cell-to-cell transfer of α-synuclein and focus our review on the long-distance axonal transport of α-synuclein along neurons.

## Introduction

Pathological accumulation of misfolded α-synuclein (α-syn), leading to cell dysfunction and cell death, plays a central role in the pathogenesis of Parkinson's disease (PD). PD is not infectious like prion diseases; however, recent studies suggest the existence of a common mechanism underlying the propagation of α-syn pathology throughout the brain 2, 24. In experimental paradigms, α-syn transferring from cell-to-cell and initiating the prion-like spread of pathology in synucleinopathies has been hypothesized. In this review, we highlight the involvement of α-syn in PD and in the progression of the neuropathology and symptoms of PD. We describe the idea of a prion-like mechanism contributing to PD pathology, discussing recent studies investigating cell-to-cell transfer of α-syn and focus on the long-distance axonal transport of α-syn along neurons. Finally, we address the evaluation of Braak's hypothesis and discuss the prion-like hypothesis for PD research.

## The Involvement of α-Synuclein in Parkinson's Disease

PD is the second most common neurodegenerative disorder. A clinically important feature of PD neuropathology is the loss of dopaminergic neurons in the substantia nigra pars compacta. It is believed that the resulting depletion of dopamine in the striatum plays a key role for the motor symptoms (eg, bradykinesia, resting tremor, rigidity and postural instability) because “dopamine-replacement” pharmacotherapy is relatively effective at reducing the motor disturbances. Recent years have, however, shown that several non-motor symptoms (eg, depression, dementia, anosmia and sleep disturbances) 19 are also a source of major morbidity, and they respond relatively poorly to dopamine replacement therapy 11. Consequently, greater attention is being paid to neurodegenerative changes outside the nigrostriatal pathway in PD. In this context, the presence of intracellular protein inclusion bodies, so-called Lewy bodies (LB) and Lewy neurites (LN), is now believed to be important. Friedrich H. Lewy (1912) 48 first described these inclusions over a century ago. It was not until 1997 when it was discovered that aggregated α-syn is the major constituent of LBs and LNs 2, 24, 30, 72 that the modern era of PD neuropathology research was born. Not only is α-syn the main component of Lewy inclusions in sporadic PD, but missense mutations (A53T, A30P, E46K) in the α-syn gene are also associated with autosomal dominant PD 19, 41, 67, 78. Furthermore, duplications and triplications in the α-syn gene lead to a severe neurological syndrome with parkinsonian features 10, 11, 70 and certain single-nucleotide polymorphisms in the α-syn gene are associated with increased PD risk 48, 56.

α-Syn protein is abundantly expressed in the brain as well as in multiple other central and peripheral tissues 4. Maroteaux *et al* 57 first described the localization of α-syn to the nucleus and the presynaptic terminal. Although the full function of α-syn is yet to be defined, it is certainly involved in vesicular trafficking and release, related to its associations with the SNARE complex proteins 9, 60. α-Syn consists of three domains: an amino-terminal lipid binding α-helix, a non-amyloidogenic core (NAC) domain and an unstructured carboxy-terminus. These three regions are necessary for the misfolding of the protein 37. α-Syn is considered natively unfolded, but its amino-terminus forms α-helical structures when bound to phospholipids. Recently, investigations of endogenous α-syn analyzed under non-denaturing conditions in cell lines and mouse brain tissue revealed that α-syn might exist as a folded, stable tetramer with a molecular weight of about 58 kDa 5, 76. These results have proven controversial 20. What is more certain is when α-syn is misfolded, the random coil of the NAC domain forms β-sheets, which participate in protofibril and fibril formation 68, 74. Importantly, two mutations in α-syn (E46K, A53T) increase the oligomeric and fibrillar forms of α-syn 47, further highlighting the importance of the aggregation of α-syn in its toxicity.

## The Braak Hypothesis

What is the connection between α-syn aggregates and the development of PD? Braak *et al* 6 suggested that in idiopathic PD post-mortem brain tissue, LB pathology appears in a stereotypic pattern depending on how advanced the disease is. In stage 1, Lewy pathology (primarily LNs) is found in the olfactory bulb (and anterior olfactory nucleus) and the dorsal motor nucleus of the glossopharyngeal and vagal nerve. In stage 2, the Lewy pathology continues to ascend toward the brainstem, reaching the medulla oblongata and pontine tegmentum. People in stages 1 and 2 do not have clinical PD but might exhibit signs of anosmia and constipation. It is unknown whether these individuals would develop PD later on. In stage 3, the pathology appears in the amygdala and substantia nigra. It is at this stage that marked nigral neurodegeneration is expected to occur and the individual will start to develop motor symptoms. Thus, it is not until Braak stage 3 that people will be clinically diagnosed with PD. The LBs, and to a larger extent LNs, are also found in the forebrain and cerebral cortex in stage 4. In stages 5 and 6, the pathology also appears initially in the anterior association and prefrontal areas of the prefrontal cortex and continues to spread toward the posterior association areas. In summary, Braak *et al* suggested that α-syn pathology is initiated in the peripheral nervous system and olfactory bulb, ascends toward the brainstem and into the midbrain, and then eventually spreads to the forebrain. Thus, if α-syn pathology starts in, for example, the gut, it spreads over very long distances in the nervous system during several years.

Because the vagus nerve connects the brainstem and the enteric nervous system, Braak *et al* concluded that PD could in fact begin in the gut and then travel retrogradely toward the brain 27. The appearance of pathology initiating in two separate locations gave rise to the “dual-hit” hypothesis 28. It was speculated that an unknown pathogen may enter the nervous system through both the olfactory bulb and the enteric plexus of the stomach 27. We, and others, now suspect that a misfolded form of α-syn might play the role of the unknown pathogen. Misfolded α-syn could spread from one cell to another and trigger aggregation of α-syn in the recipient cell. One requirement for this mechanism is the ability of α-syn to travel from cell-to-cell as well as to move from one brain region to another distant structure, acting like a “long distance runner.”

## Cell-to-Cell Transfer: Evidence from Human Grafting Studies

Mounting evidence points toward α-syn acting as a prion-like protein. By suggesting that α-syn is a prion-like protein, we mean that misfolded α-syn could be responsible for the intercellular transmission of PD pathology. We are not implying that the disease can be transmitted between individuals 31. Initial evidence supporting the process in humans became apparent from patients who received embryonic neural tissue grafted into the striatum to replace lost nigral dopaminergic neurons. Autopsy revealed that these young transplanted neurons, introduced stereotaxically into the host brain over a decade prior to death of the patient, contained α-syn pathology 38, 40, 42, 49, 50. One possible explanation for the presence of LB in young transplanted neurons is that α-syn can transfer directly from the host brain to grafted cells 7. The question is then, did α-syn transfer from the host to seed the aggregation of endogenous protein in the grafted cells? Subsequent to the finding of LB pathology in grafted embryonic tissue, model systems have been developed to examine both the transfer and the seeding of α-syn *in vitro* and *in vivo*.

## Cell-to-Cell Release, Uptake, Transfer and Seeding of α-Synuclein: Evidence from *in vitro* and *in vivo* Studies

**Figure 1:**
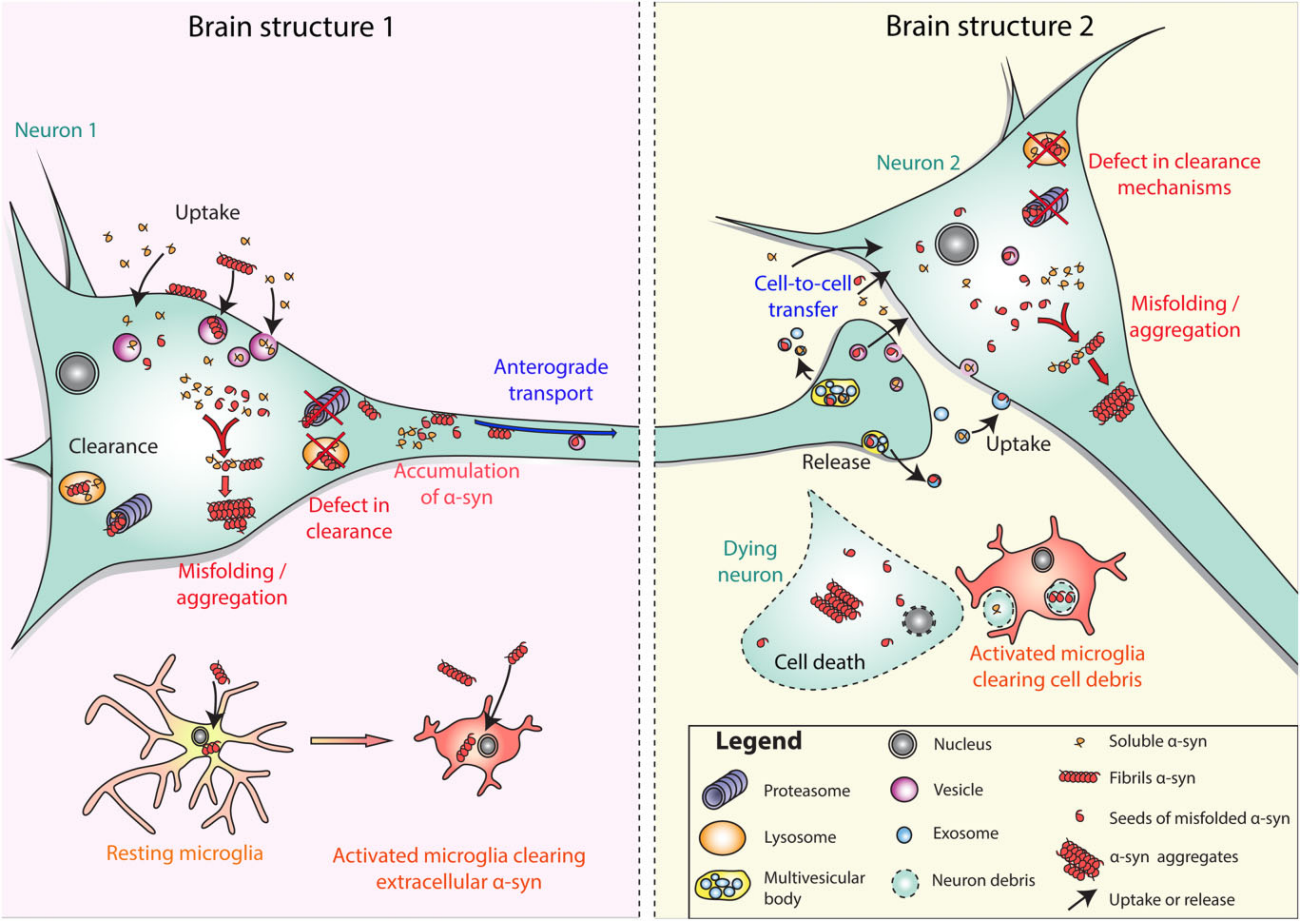
*Schematic presentation of the possible mechanisms underlying the spread of synuclein pathology and α-syn aggregation in Parkinson's disease (PD)*. In the brain structure on the left, conditions of cellular stress cause α-syn to misfold within the neuron (neuron 1), or misfolded α-syn is taken up from the extracellular space. Internalized misfolded α-syn might be degraded by clearance mechanisms such as the ubiquitin proteasome system, lysosomes and autophagy. Under particular conditions of stress and/or clearance failure, misfolded α-syn might not be effectively degraded. Thus, the remaining misfolded α-syn might recruit soluble α-syn in a seeding mechanism, thereby converting it into misfolded protein, initiating aggregation within neuron 1. The remaining misfolded α-syn may also undergo intracellular axonal transport, via fast axonal transport or via slow component b axonal transport within the axon of neuron 1. At the terminal of neuron 1, which is located in brain structure 2, transported (misfolded) α-syn might be released by exocytosis, or in exosomes. The α-syn released by exocytosis or in exosomes can then be taken up by the surrounding neurons such as neuron 2 (cell-to-cell transfer). The same cascade of events including recruitment of endogenous α-syn, seeding and aggregation, clearance and then failure of clearance is proposed to lead first to the formation of α-syn aggregates (neurons 1 and 2), and in the end, to the death of the host neuron (dying neuron). Misfolded α-syn released into the extracellular space from living neurons or dying cells can activate microglia that take up and degrade misfolded α-syn.

The hypothesis of prion-like transmission of α-syn pathology relies on the premise that a sick neuron could release its α-syn or that α-syn gains access to the extracellular space when the neuron dies. Once in the extracellular space, the misfolded α-syn could then be free to enter an adjacent neuron and act as a template, seeding the aggregation of numerous α-syn monomers and initiating the formation of a LB or LN 25 (Figure 1).

### The release of α-synuclein

The first step in this hypothesis requires that there is cellular release of α-syn. Under physiological conditions, small amounts of α-syn are released from cells in the absence of membrane damage, despite the lack of a secretory peptide sequence on α-syn 44. Endogenous monomeric and aggregated forms of α-syn can be secreted into the culture medium of differentiated human neuroblastoma cells and rat primary cortical neurons 14, 24, 44 and are detected in human cerebrospinal fluid and plasma 17. The secretion of α-syn maybe due to its association with vesicle trafficking as α-syn can induce the aggregation of yeast Rab GTPase proteins and block endoplasmic reticulum-Golgi trafficking 12, 23, 71. Release of α-syn from neurons is partially mediated by exocytosis and is increased under conditions of misfolded proteins 24, 32, 44, 45, 46, 63, 75. Also, α-syn can be released from enteric neurons in a neuronal activity-regulated manner 63. α-Syn is secreted by non-classical exocytic or endocytic pathways 44. α-Syn can be directly integrated into secretory vesicles and subsequently released by exocytosis or be translocated to early endosomes 1, 18. From early endosomes in neuronal cultures, α-syn protein can either be released to the extracellular space through the recycling endosome or incorporated to intraluminal vesicles of multivesicular bodies 26. Multivesicular body cargo, including α-syn, can be directed for degradation by fusion with lysosomes or secretion by fusion with the plasma membrane, upon release of exosomal vesicles 26.

### Uptake of α-synuclein

The ability of cells in culture to take up α-syn is dependent on the cell type and the species of α-syn 13, 18, 44. In a cell culture study investigating α-syn uptake via endocytosis, higher order oligomers were the species of α-syn that entered cells more readily than monomers, highlighting the importance of α-syn oligomerization in cell-to-cell propagation 13. In cultured neuronal cells, both oligomeric and fibrillar forms of α-syn are internalized through endocytosis and targeted to the lysosome for degradation 24, 46, 54, 61, 75, 77 (Figure 1). Further support for endocytosis as a mechanism of α-syn uptake is demonstrated in studies employing endocytosis inhibitors 24, 45. For a thorough review on the uptake of α-syn into cells via endocytosis, please see 16.

### The transfer and seeding of α-synuclein

The *in vivo* data supporting cell-to-cell transfer and seeding of α-syn pathology are striking. Several groups have succeeded in producing models that display transfer of α-syn from the host brain to grafted cells similar to that hypothesized to occur in PD patients 39, 49. Desplats *et al* transplanted mouse neuronal progenitor cells into the hippocampus of transgenic animals overexpressing human α-syn 15 and discovered that 1-week post-injection, grafted cells had human α-syn immunoreactivity, and by 4 weeks, some of the grafted cells contained aggregates, which expressed human α-syn. In another study, wild-type mouse nigral tissue was grafted into the striatum of transgenic mice overexpressing human α-syn, and small amounts of host-derived human α-syn were observed in around 1% of the grafted dopamine neurons 24. Evidence for transfer and seeding of endogenous α-syn was presented 24. In a next step, rats were engineered to markedly overexpress human α-syn in the nigrostriatal pathway and then transplanted with embryonic rat nigral tissue. In this case, over 20% of the grafted dopamine neurons displayed host-derived human α-syn. Labeling with species-specific α-syn antibodies revealed evidence that the imported human α-syn seeded aggregation of endogenous rat α-syn in these naïve neurons 2. Thus, sometimes, a small area immunoreactive for human α-syn was enveloped by a larger area immunoreactive for rat α-syn derived from the host neuron 2.

To further investigate the phenomena of α-syn propagation, Kordower *et al* 39 grafted fetal rat brain tissue into 6-hydroxydopamine lesioned adult rats. After the transplant, viruses containing human α-syn were injected distal to the grafted cells. Close examination revealed a number of grafted neurons expressing human α-syn. Transferred α-syn can therefore not only propagate from neuron to neuron but can also be modified, aggregate and form pathogenic species. For a more in-depth description of the concept of cell-to-cell transfer in PD and the subsequent seeding of α-syn aggregates, please see the recent review by Dunning *et al* 62.

Transferred α-syn can play a pathogenic role. In young transgenic (A53T) α-syn mice inoculated intracerebrally with brain tissue from old transgenic (A53T) α-syn mice, early signs of motor impairment were detected. This sign of disease was associated with insoluble phosphorylated α-syn and dystrophic neurites in the raphe nucleus and the lateral vestibular nucleus in these animals 58. Importantly, inoculation with old transgenic brain tissue decreased lifespan with death occurring significantly earlier than in transgenic mice inoculated with brain homogenate from young healthy transgenic mice. In contrast, wild-type mice lacking the α-syn locus, inoculated with brain homogenate from old transgenic (A53T) mice, survived the longest 58.

The most recent developments in the field of α-syn transfer and seeding are from Virginia Lee, John Trojanowski, and colleagues. In their first paper of 2012, the authors injected recombinant human α-syn preformed fibrils or brain lysate from symptomatic transgenic mice overexpressing A53T α-syn [M83 mice, 22] into the cortex and striatum of asymptomatic transgenic mice. Induction of α-syn pathology in recipient mice as early as 30 days post-injection was observed and the pathology progressively spread to interconnecting brain regions 55. The site of injection produced differential patterns of α-syn pathology in the recipient mice. The pattern was consistent with the neuronal connections to and from the site of injection. Taking their findings a step further, Luk *et al* demonstrated transfer and seeding of α-syn using synthetic preformed fibrillar mouse α-syn injected into wild-type mice 53. In this study, preformed fibrils assembled from recombinant mouse α-syn were injected into the dorsal striatum. Phosphorylated α-syn (indicating recruitment of endogenous α-syn that had undergone post-translational modification) was observed in neurons at the injection site 30 days post-injection. The authors also described LB-like structures containing α-syn in some brain regions interconnected with the striatum, such as the neocortex. However, there was no phosphorylated α-syn in brain regions that do not project directly to the striatum, suggesting that, in fact, there was no trans-synaptic transmission of α-syn aggregation in this paradigm. Notably, dopaminergic neurons in the substantia nigra, which are one population that project to the injection site, frequently exhibited α-syn aggregates. The consequences of these changes were striking. The accumulating α-syn led to a gradual loss of dopaminergic cells and depletion of striatal dopamine, accompanied with motor deficits on the rotarod and wire hang test 53. In agreement with the study by Mougenot *et al* 58, injections of preformed α-syn fibrils did not give rise to α-syn aggregates when injected into α-syn null mice, confirming that recruitment of endogenous α-syn is crucial for the pathogenic process.

## Crucial Factors Affecting the Capacity of α-Synuclein to Act in a Prion-Like Fashion

Based on the *in vitro* and *in vivo* studies described earlier, what factors affect the capacity of α-syn to act in a prion-like fashion? Clearly, several steps are crucial in the process. For example, for α-syn to be an effective prion-like protein, it has to be released by cells; not be cleared and degraded by microglia; but taken up by neighboring neurons; evade intracellular protein degradation and promote seeding of aggregates in the cytosol, and importantly, undergo long-distance transport from one brain structure to another region so that the neuropathology can spread over long distances.

Recent immunization studies in α-syn transgenic mice indicate that antibodies targeting α-syn will promote its uptake and degradation by microglia and thereby reduce the likelihood of intercellular α-syn transfer 3. As expected, studies on intracerebral injections of α-syn indicate that both the molecular species of α-syn and the α-syn protein homeostasis in the mice receiving the injections will influence the outcome. Luk *et al* 53 used recombinant mouse α-syn in their studies on intracerebral injections of α-syn. Not only was the protein synthetic, the fibrils were also sonicated to create a mixture of very small seeds of fibrillar α-syn (please see the electron micrographs in supplementary Figure 1B in reference (53)).

The efficacy at which monomeric, oligomeric or fibrillar forms of α-syn are taken up by cells vary in cell culture 45 and the same most probably applies *in vivo*, too. This should significantly impact the likelihood of pathological aggregates forming inside the neurons in the injected brains. Although monomeric, oligomeric and fibrillar forms of α-syn can all be taken up by cells from the extracellular space, and the uptake of monomers is efficient 24, in the study by Luk *et al*, monomers did not induce pathology 53. It was not determined whether the injected monomeric α-syn was still present in the brains after 30 days. It is likely that the monomeric α-syn was taken up, transported away from the injection site and potentially cleared by the first (30 days) time point investigated. Alternatively, the injected monomeric α-syn was impossible to distinguish from the host protein because mouse recombinant α-syn was injected into mice 53. In the studied paradigm, only aggregated α-syn would be possible to detect and as an increased load of monomeric α-syn apparently was not sufficient to promote aggregation, this readout was negative.

The studies on injections of synthetic preformed fibrillar mouse α-syn in wild-type mice demonstrate that the presence of endogenous α-syn is crucial in the seeding of aggregates 53, 58. Thus, α-syn knock-out mice did not develop pathology when preformed α-syn fibrils were injected. Furthermore, the degree of homology between the “α-syn seed” and the α-syn in the recipient neuron can be important. Human α-syn shares 95.3% homology with mouse α-syn, which carries the native rodent sequence 43. Perhaps the rodent sequence of α-syn is more permissive to seeding and aggregation 51, 59. It would be interesting to investigate how well mouse and human fibrillar α-syn species interact, directly comparing the time delay in cellular uptake and the lag time to produce α-syn aggregate pathology as well as the transport of these species to interconnected brain regions *in vivo*.

## Axonal Transport of α-Synuclein: is α-Synuclein the Long Distance Runner?

### α-Synuclein is transported via fast and slow component axonal transport

α-Syn is actively transported in both directions in axons. Proteins are transported along axons via either the fast transport component (FC, 100–400 mm/day), slow transport component a (SCa, 0.1–2 mm/day) or slow transport component b (SCb, 2–10 mm/day) 29, 69. Similar to other proteins, α-syn can be transported along axons 35, 36, 52, 73. A study utilized rat visual pathways as a model system 36 and found that approximately 25% of wild-type α-syn travels using FC axonal transport, presumably after binding the membranes of vesicles. A majority (approximately 75%) of rat α-syn is transported in the SC (approximately 15% in SC a and approximately 60% in SC b) 36. According to studies in mouse hippocampal neurons, this α-syn is transported slowly along axons to synaptic terminals 73.

Reports on the effects of point mutations in α-syn on its propensity to undergo axonal transport are conflicting. Mutant A30P α-syn does not undergo the same FC axonal transport as the wild-type form in the rat visual system, possibly due to its reduced membrane binding 36. Another study investigating the axonal transport of α-syn in peripheral nerve demonstrated that the transport of human and mouse α-syn is not affected by the A30P and A53T α-syn mutations 52. With increasing age, however, the rate of α-syn axonal transport decreases 52.

### α-Synuclein is transported along axons in both directions in the central nervous system

**Figure 2:**
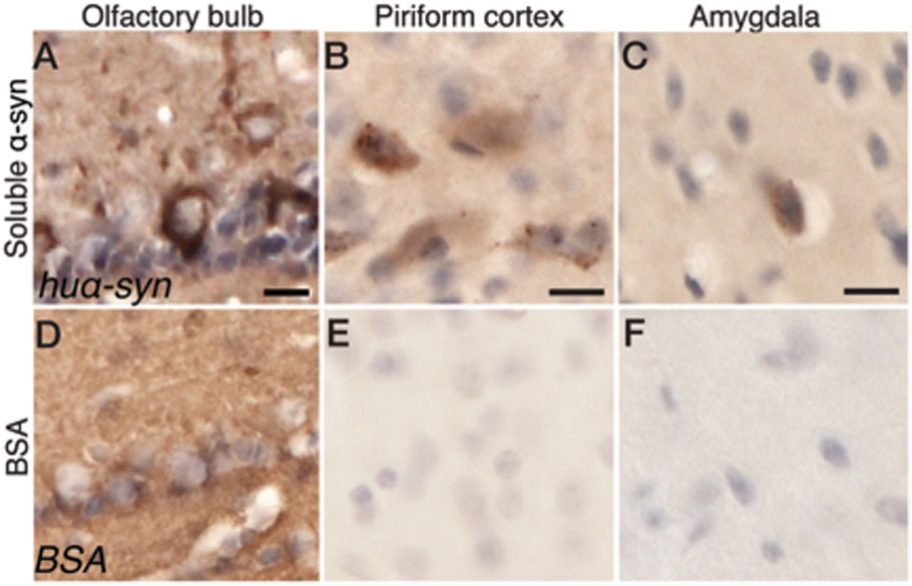
*Uptake of α-syn in the olfactory bulb and transfer of soluble α-syn to interconnected regions in the mouse brain*. Human α-syn staining in the injected olfactory bulb **(A)**, the ipsilateral piriform cortex **(B)** and the ipsilateral amygdala **(C)** after injection of soluble α-syn. Bovine serum albumin (BSA) staining in the injected olfactory bulb **(D)**, the ipsilateral piriform cortex **(E)** and the ipsilateral amygdala **(F)** after injection of BSA. The scale bar represents 10 μm. (Data from Rey *et al*. 2013, submitted.)

Cell culture studies using microfluidic chambers to isolate and separate neuronal cell bodies from their terminals have demonstrated that the α-syn that is taken up can also be transported along the axons of neurons. In mouse primary cortical and hippocampal neurons, α-syn can travel in both retrograde 14, 75 and anterograde directions 21, 75 (Figure 1). Recently, we studied the fate of various molecular species of human α-syn (soluble α-syn or fibrils) after injection into the olfactory bulb of wild-type mice. We asked whether α-syn is transferred to interconnected structures within a few hours after injection. As a control protein, we injected bovine serum albumin (BSA). We detected the proteins we injected using antibodies that recognize specifically human α-syn (syn211) or BSA (Figure 2). Within 20 minutes, human α-syn was taken up by mitral cells, which are the relay cells of the olfactory bulb that project to other olfactory structures. Less than an hour after injection, we found injected soluble α-syn was present in multiple structures directly connected to the olfactory bulb, for example, piriform cortex and amygdala. Consistent with the *in vitro* studies mentioned earlier, our detailed anatomical analyses suggest that the α-syn we had injected was transported both in anterograde and in retrograde directions. By contrast, our control protein, BSA, was rarely taken up by olfactory bulb cells and did not transfer to other brain structures, suggesting that soluble forms of α-syn are both exceptionally good and fast long distance runners.

### Can α-synuclein be transported along peripheral nerves from the gut to the brain?

Is there any evidence that misfolded α-syn can undergo long-distance transfer from the gut to the brain, in keeping with Braak's proposed dual-hit hypothesis? A recent study explored in some anatomical details how misfolded α-syn might spread from the enteric nervous system to the central nervous system 65. Pan-Montojo *et al* followed up their earlier report suggesting that following mitochondrial inhibition, by giving rotenone locally in the gut, α-syn levels are increased in enteric neurons and aggregated protein is released 64. Eventually, this misfolded α-syn reaches the central nervous system where it was suggested to cause degeneration of dopamine neurons accompanied by motor deficits 64. They suggested that it is the sympathetic and parasympathetic nerves that take up the extracellular α-syn and retrogradely transport it to the soma of the autonomic nervous system neurons. In this most recent study, the investigators severed the sympathetic and parasympathetic nerves in the same animal model of PD 65. Resection of sympathetic and parasympathetic nerves halted the progression of PD-like α-syn pathology to the previously connected neurons within the intermediolateral column of the spinal cord, the vagal dorsal motor nuclei and the substantia nigra. The claim that α-syn can be transported along long autonomic nervous system structures supports the notion that α-syn is a long distance runner with the ability to spread pathology from one structure to another.

## An Evaluation of the Braak Staging

The Braak hypothesis that Lewy pathology spreads throughout the brain in regions that are connected to the peripheral nerves or the olfactory system is not uncontroversial. One reason why the hypothesis remains contentious is partly due to the fact that Braak staging and severity of PD symptoms do not always correlate 8. Certainly, some individuals with relatively severe α-syn pathology discovered post-mortem were never diagnosed clinically with PD prior to death 66. Moreover, in some PD cases, the distribution of Lewy pathology does not match any of the Braak stages 33, 34. In Braak's stages 1 and 2, lower brainstem synucleinopathy is designated to represent “early” PD. However, criticism arose as to whether these individuals would develop PD later on 8. When considering these cases, it is important to note that Braak's staging uses the presence of LBs and LNs as the identifiable hallmark. Possibly, smaller aggregates of α-syn that do not qualify as LBs or LNs contribute to the variation in Braak's staging in some cases.

What else could explain the differences between the findings of Braak and some other investigators? Notably, the methodology varies between different studies. Braak *et al* 6 used 100-μm-thick sections sampling greater volumes than in commonly used 10-μm-thick sections. Differences in post-mortem time before tissue fixation and variations in fixation and immunostaining protocols are other confounders. The clinical data on neurological and psychiatric symptoms prior to death can also be inconsistent between studies. Until the technology exists to definitively image insoluble α-syn longitudinally in living people, it is impossible to evaluate fully the Braak hypothesis.

## Conclusions

The spreading of α-syn pathology in PD, as suggested by Braak *et al*, would require that α-syn can travel along long unmyelinated axons and seed aggregation in new neurons at the destination during a slow process that takes years or even decades. To date, no study has unequivocally demonstrated α-syn transport from one brain structure to another in a living human or animal. The development of *in vivo* imaging techniques to visualize the movement of different molecular forms of α-syn would help us address the Braak hypothesis and understand if α-syn can act like a prion-like protein in PD. Assuming that this is the case, identifying the mechanisms of cell-to-cell spread and transport of α-syn might allow for the development of agents to block these processes. Such agents could represent a new generation of therapeutics in the fight against PD and provide the first truly disease-modifying agents that can slow the progression of PD.
